# Summer Complaint

**Published:** 1896-06

**Authors:** Paul J. Barcus

**Affiliations:** Crawfordville, Ind.


### Selections and Abstracts.

---

### SUMMER COMPLAINT.

By PAUL J. BARCUS, M. D. Crawfordville, Ind.

The diarrheal diseases of infancy for the last two years have been somewhat neglected by the general profession, but not because we are, as a general of old was, when he wept because there were no more worlds to conquer, for it is an indisputable fact that the present status of our knowledge of the etiology and our present treatment of many of these diseases are largely empirical and unsatisfactory.

Although many things that ten years ago were obscure to us are plain now, and our treatment of certain classes of these diseases is on an entirely different basis, and the study of many of these maladies must be from a different standpoint; yet the classification of the bowel troubles of infancy is as it has been for years. It is a surprising fact that the latest text-books on diseases of children, with but few exceptions, treat of the bowel troubles in much the same manner as years ago, and if you will examine your libraries, you will find that each writer has an original way of classifying these maladies. While this may be commendable in the writer, as serving to impress his individuality upon his works, it is very harassing to the student, and not conducive to a clear understanding of these maladies by the general practitioner.

This subject has been before the American Pediatric Society for two years, and its committee on revision of the nomenclature of the diseases of the gastro-enteric tract will report at the meeting te be held the last of this month.

The older classification of all the bowel troubles was upon a pathological basis,, while recent investigation has demonstrated that symptomatically there is but little difference between many lesions of different portions of the intestinal tract, and many fatal cases of summer complaint reveal no lesion of the lining membrane of the bowel whatever. Many of the symptoms that our teachers were fond of crystallizing into our memories as pathognomonic of certain conditions co-existing within the bowels, recent investigation has demon strated to be misleading and unreliable.

The terms diarrhea and dysentery are about to occupy symptomatic relations in the nomenclature of disease.

So with cholera infantum, which is a severe attack of summer complaint. The term is useless, for it lies with each individual to draw the line where ordinary summer complaint shall end and cholera infantum begin, if it is given a special classification. In our judgment any classification of the diarrheal diseases of infancy and childhood that is based on other than etiological grounds is unscientific and impracticable from a working standpoint.

Much the best classification that we have seen is that of Christopher, of Chicago. He divides the causes of diarrhea into four classes:

1. Causes arising in tissue change, involving disturbance of nutrition.
2. Causes arising in poisons developed in the blood.
3. Poisons developed in or on the intestinal wall.
4. Poisons developed in the intestinal contents.

Summer complaint comes under the last head in this classification, and is the best term by which to designate a class of bowel troubles of infancy that occur mostly during the summer months, due to the presence of micro-organisms, that in the process of fermentation generate chemical poisons that are capable of producing diarrhea, often fever, depressed heart-action, renal congestion and disturbance of the nervous system in the way of convulsion and coma. It commences as soon as the atmospheric temperature has a daily minimum of 60 degrees, because this is the degree of heat required for the bacteria to proliferate; advances in severity as the warmest season approaches, and declines as the cool weather approaches in the fall.

There are three essentials to its production: (1) bacteria, (2) proper heat, (3) proper culture medium or food for the bacteria. These factors are rendered more potent by innumerable circumstances, chief of which are bad hygienic surroundings, weaning, improper feeding as to regularity, amount and kind, tender age of patient, constitutional weakness, and other diseases.

The presence of micro-organism in the human feces has been known for almost two centuries, but little definite knowledge has been acquired of the morphology of these bacteria, or of their life histories, until within the past fifteen years. But a few years ago it was discovered that within a few hours after birth bacteria are found in the intestinal contents, and two forms were ascertained to be constantly present: the bacteria lactis ærogenes and b. coli commune. These two are so far in excess of all others that such other forms as may be present in health are regarded as accidental and depend upon the diet of the host. Milk diet is capable of supporting but a limited variety of bacteria, and if the stools are filled with multitudes of other forms, incidental to a mixed diet, a restriction to milk alone will starve out all others, or leave but few, save the two before mentioned. Vaughan, of Ann Arbor, to whom the profession is greatly indebted for his researches in this line, especially into the nature of milk infection, has produced diarrhea and vomiting with nervous disturbances in the lower animals, by means of the chemical poisons obtained from pure cultures of several forms of bacteria, isolated from the stools of infants suffering from summer complaint.

Physiological experimentation upon the lower animals and autopsies upon cases of summer complaint that have suffered from severe diarrhea with convulsions and great depression have demonstrated that there are no pathological lesions in the bowel, brain or anywhere else to account for the violent symptoms, and we are forced to the conclusion that these symptoms are due to the chemical products of fermentation.

These poisons have been demonstrated to be numerous and of distinct chemical compositions, showing that the disease is not due to a single cause, but that there are probably many separate forms of bacteria that when present in the bowels under favorable circnmstances are capable of generating a poison that will produce all the symptoms of summer complaint. No one form has been found to be uniformly present, and no less than forty different varieties have been isolated, pure culture of many of which will generate poisons that will produce vomiting, diarrhea, nervous twitchings and convulsions when administered to the lower animals.

These ptomaines are of nitrogenous composition, and it follows that a nitrogenous diet in summer complaint is followed by the most pronounced symptoms. A few years ago it was stated by Escherich that if albuminous decompositions, with very foul, offensive stools, be present, albuminoids should be withheld and carbo-hydrates given. If acid fermentation exist, with sour, but not putrefactive stools, the carbo-hydrates are to be withheld and an albuminous diet prescribed.

While Baginsky claims to find this plan unsatisfactory after trial, many of the most eminent American physicians are teaching this to-day. We believe the theory to be correct in the initial stage of many cases. But we find but few cases that are confined to either an albuminous or carbo-hydrate diet. And as stated by Baginsky, the stools are not reliably symptomatic of what is going on in the bowels.

As pointed out by Holt, of New York, there is a preliminary dyspepsia in the vast majority of cases of summer complaint. The disease being of bacterial origin, and the proliferation of these bodies in the intestinal tract at a minimum, and limited to a few varieties so long as digestion and absorption are healthy—so digestion and absorption must be imperfect before there can be a dangerous increase in the number of the disease-producing bodies—barring cases in which there is infection by means of the ingestion of bacteria in overpowering numbers.

The pathology depends upon the duration and virulency of the disease. In cases that have succumbed in a few hours no lesions are found anywhere. In those of longer duration the membrane lining the bowel becomes ulcerated and the exudation upon the surface of the ulcers affords food for the bacteria, and in this manner the system itself keeps up the manufacture of the poisons on the surface of the mucuos membrane of the bowel.

The etiology suggests the treatment. The first essential is to regulate the diet; starve out the bacteria by shutting off their food supply. Many cases require but little else than a regulation of the diet, and this is usually sufficient in preliminary dyspepsia.

In the way of prophylaxis parents should be instructed in the methods of sterilizing foods, especially milk. In severe cases the milk should be shut off as well as all other foods, the duration of abstinence depending upon the severity of the case. The next indications are to rid the bowels of the fermenting contents, to overcome the poisonous effects of the toxines evolved, and prevent their further formation.

To obtain the first, nothing else is so good as calomel, prescribed in an initial dose of from gr. ss–i ss, to be followed every two hours by gr. $\frac{1}{8}$–$\frac{1}{12}$, until the effect of the laxative is obtained.

To still further cleanse the bowel it should be cautiously irrigated, for the same reason that we wash out a septic uterus in peurperal septicemia. The bowel should be filled to the cecum, for autopsies have shown that this is the point of entrance of the toxic elements in nearly all cases.

The technique of intestinal irrigation is a matter of great importance, and should not be entrusted to an inexperienced nurse. Thoroughness of the work may be facilitated by manipulation in the course of the large bowel, enabling the water to wash out the large sulci which the laxative may pass over, and which may contain the fermenting mass that is causing the trouble.

When there is evidence of profound impression upon the nervous system, be it manifested by exalted muscular activity or depression, the careful exhibition of opium or belladonna dulls the sensibility of the nervous centers, stimulates the patient and temporarily sustains life. At the same time means should not be neglected to reduce high temperature or combat depression.

In cases of uncontrollable vomiting it may become necessary to wash out the stomach, but this is rare. When attended with great loss of the watery elements of the blood, injections have been recommended of normal salt solution into the tissues of the thigh, or in desperate cases into the peritoneal cavity with antiseptic precautions.

The intestinal tract having been cleansed by the laxative and the large bowel washed by irrigation, this state of cleanliness must be maintained by the systematic use of irrigation, and by the administration of antiseptic remedies. Of these the chief are calomel, bismuth and salol.

Calomel has given us the best results in gr. $\frac{1}{20}$-$\frac{1}{12}$ doses every hour in foul-smelling stools, with no great waste of the watery elements.

Bismuth is unquestionably the best therapeutic agent in all cases in which there is any considerable lesion of the bowel and in large watery evacuations, but to be efficient it must be administered in large doses.

Salol in prolonged cases in which there is evidence of ulceration of the first part of the small bowel, care being used to avoid carbolic acid poisoning. In prolonged cases, probably the best results are obtained by the systematic daily use of irrigation, with proper feeding.

The selection of the most suitable drug for a particular case must, to a large extent, be empirical, and depend upon an array of circumstances, not the least of which is the method of the physician in charge. With the greatest care, a large per cent. of these little ones disappoint our expectations, and we are reminded that the treatment of summer complaint is yet unsatisfactory. There is much to be done by the bacteriologist and chemist before the clinician can outline a uniformly successful method of treating these maladies. In the light of the advancement that has been made in the few years, since these cases were taken out of the hands of the grandmothers, we look for better and better results, as we pave the way for better work by endeavoring to educate mothers in the methods of feeding their infants, snd to the necessity for calling medical aid in the early stages of these diseases.

